# Supplementary material for: Elevated H3K79 homocysteinylation causes abnormal gene expression during neural development and subsequent neural tube defects
Source: Nat Commun. 2018 Aug 24;9:3436. doi: 10.1038/s41467-018-05451-7 (PMC6109101; doi:10.1038/s41467-018-05451-7)
Supplement: Supplementary file 1 — Supplementary Information [file 41467_2018_5451_MOESM1_ESM.pdf]

Title: Elevated H3K79 homocysteinylation causes abnormal gene expression during neural development and subsequent neural tube defects

First author: Zhang,[1], Bai, [1], Mei,[1] , et al.

Supplementary Figures

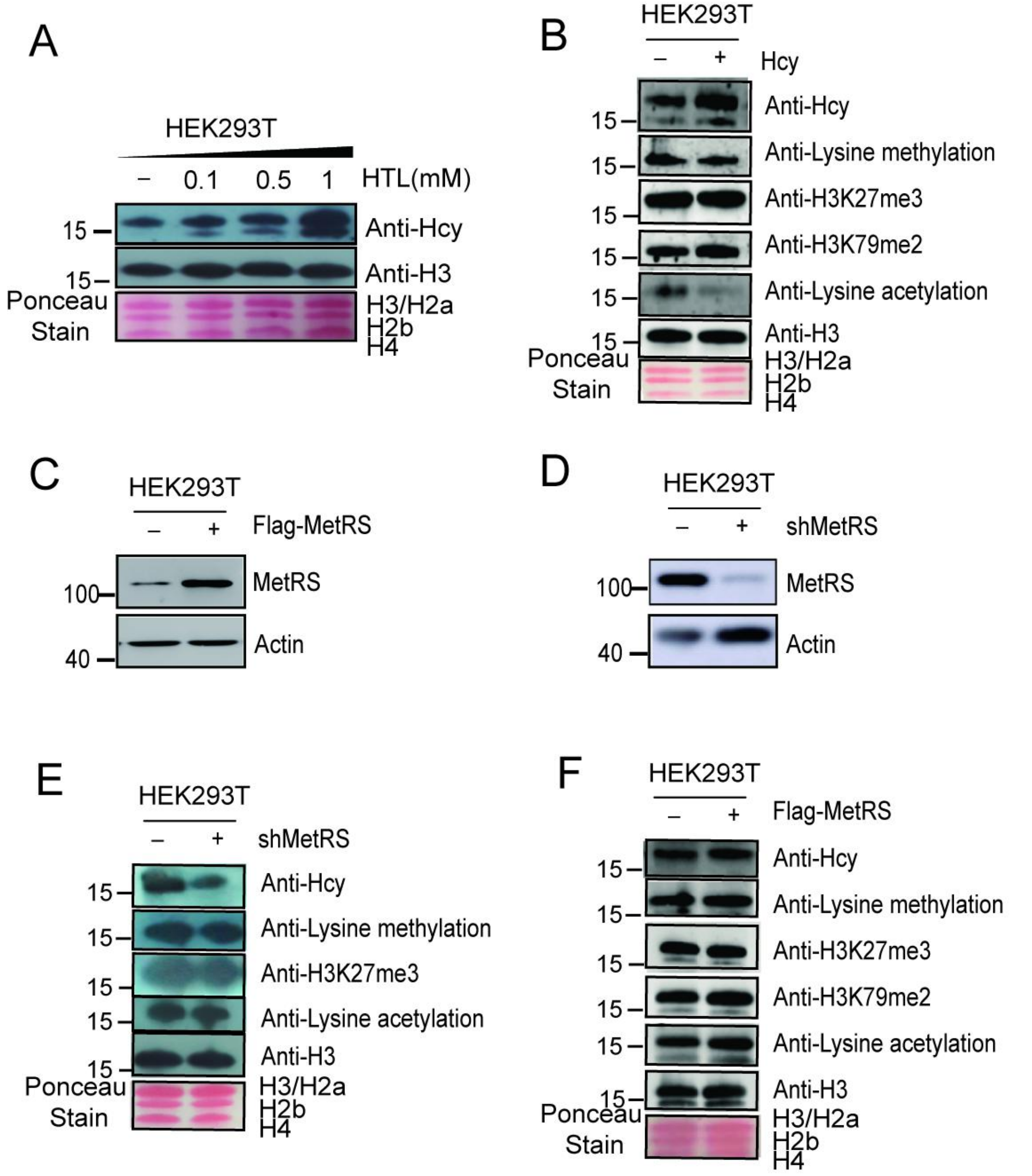

Supplementary Figure 1. Levels of Hcy or HTL affect histone homocysteinylation in HEK293 cells and MetRS-dependent.

(A) Western blotting of histone 3 Hcy modification from HEK293 cells treated with different concentrations of HTL for 4h.
(B) Western blotting of cell lysates from HEK293 cells treated with 0.5mM Hcy for 4h
(C) Western blotting of cell lysates showed MetRS level from HEK293 cells transfected with or without MetRS plasmid for 36h.
(D) Western blotting of cell lysates showed MetRS level from HEK293 cells with or without MetRS knockdown.
(E) Western blotting of cell lysates from 0.5mM HTL-treated HEK293 cells with or without MetRS knockdown.
(F) Western blotting of cell lysates from HEK293 cells transfected with or without MetRS plasmid for 36h.

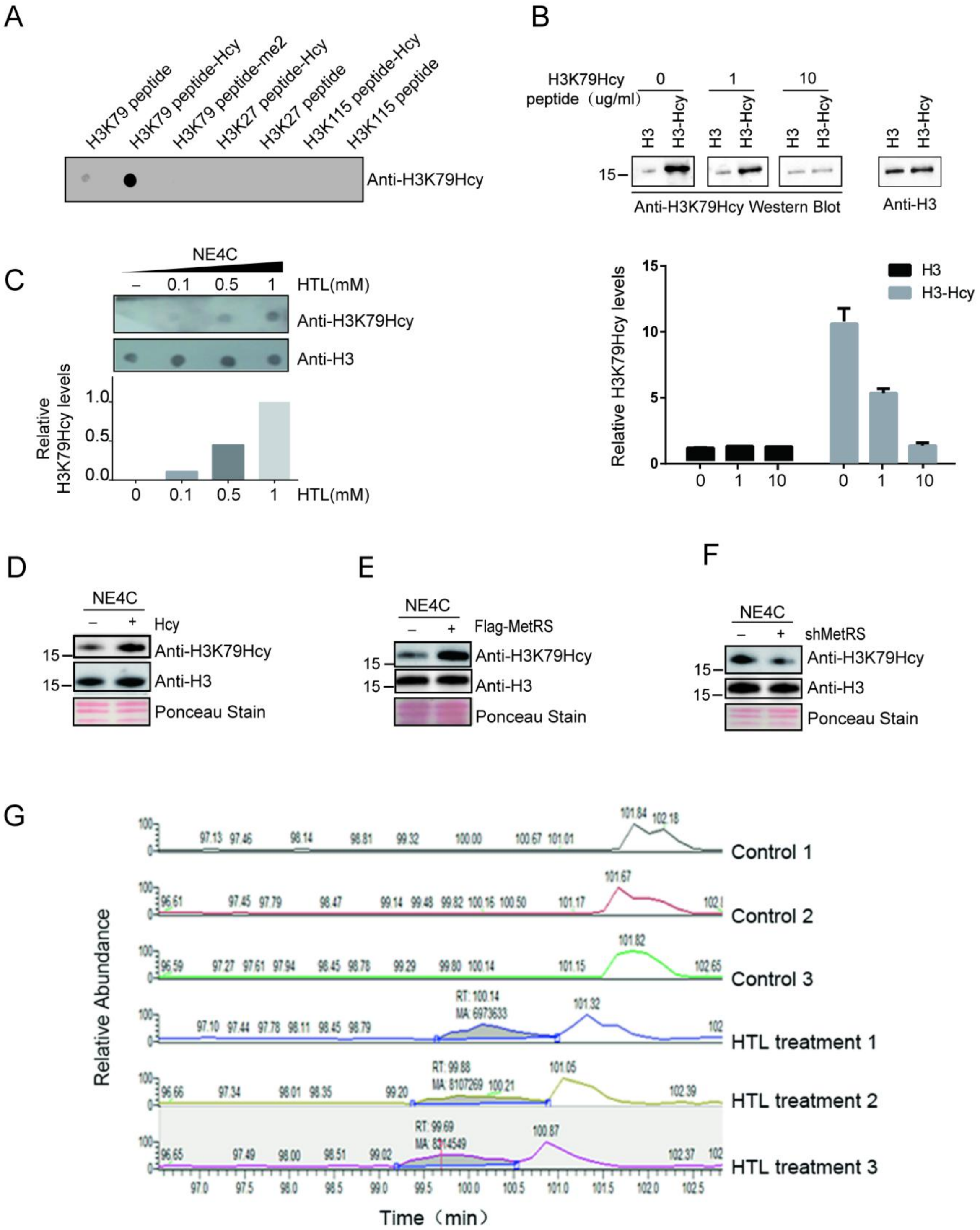

Supplementary Figure 2. Levels of Hcy or HTL regulate modification of H3K79Hcy in NE4C cells.

(A) Verification of anti-K-Hcy antibody. Specific western blotting of H3K79Hcy modification from peptides .
(B) Verification of anti-K-Hcy antibody. The homocysteinylation levels of H3 and Hcy modified H3 (H3-Hcy) were detected with anti-H3K79Hcy antibody under presence of 0, 1 or 10 μg/ml of H3K79Hcy peptide (H3K79Hcy peptide as competitor).
(C) Verification of anti-K-Hcy antibody. Western blotting of H3K79Hcy modification with increased HTL treatment in NE4C cells.
(D) Western blotting of H3K79Hcy modification from NE4C cells treated with or without 0.5mM Hcy.
(E) Western blotting of H3K79Hcy modification from NE4C cells transfected with or without MetRS plasmid for 36h.
(F) Western blotting of H3K79Hcy modification from 0.5mM Hcy-treated NE4C cells with or without MetRS knockdown.
(G) HPLC-MS/MS label-free diagram of area of H4K59hcy peptide from NE4C treated with or without 0.5mM HTL for 4h.

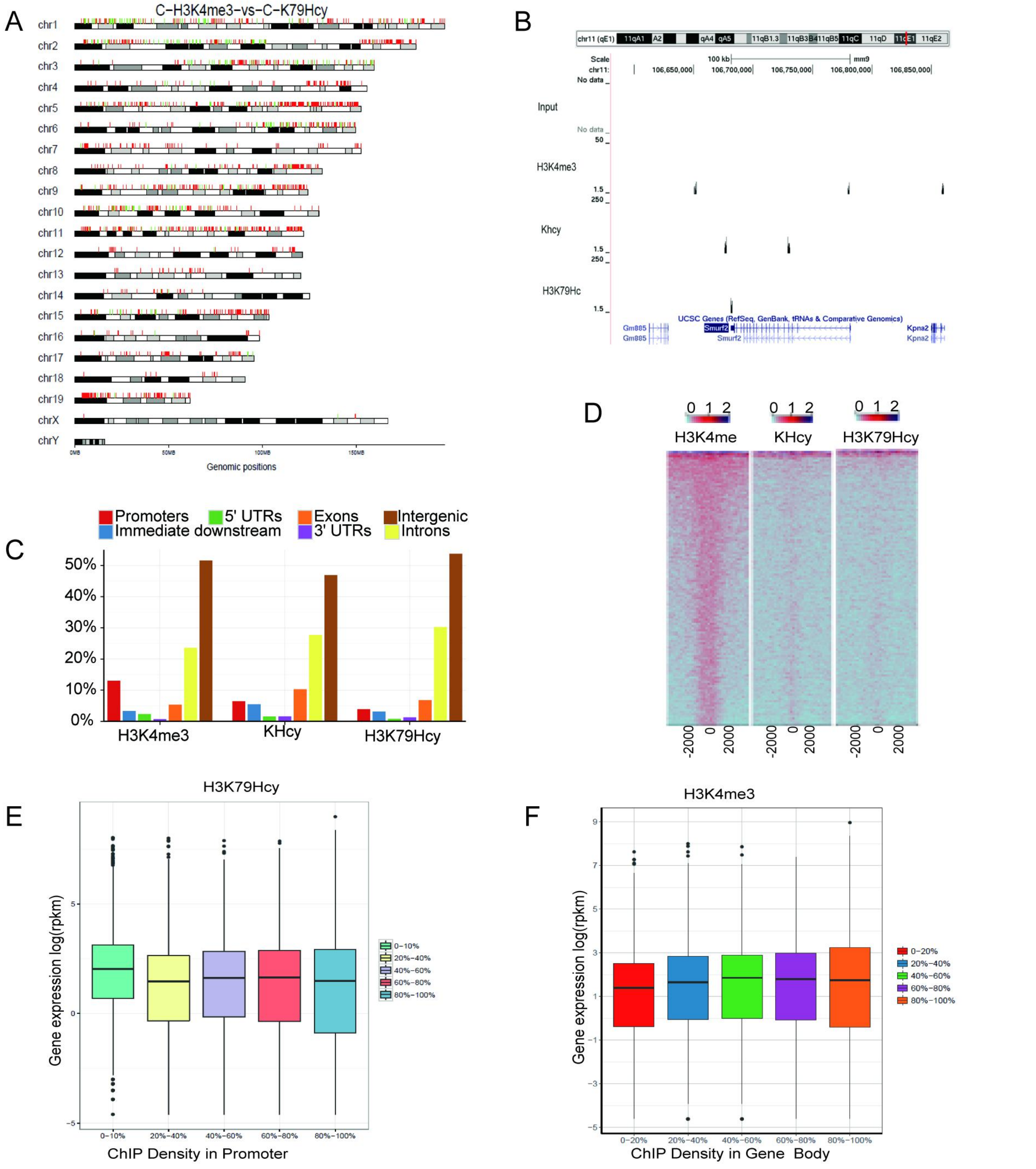

Supplementary Figure 3. ChIP-seq data analysis of H3K79Hcy and H3K4me3

(A) The difference enriched peak between H3K4me3 group and H3K79Hcy group .
(B) ChIP-seq snapshots of in put, H3K4me3 ,KHcy andH3K79Hcy in NE4C cells
(C) Chsomosome region distribution of input, H3K4me3 ,KHcy andH3K79Hcy in NE4C cells. Promoters: Peaks that reside 2000 bases upstream from gene start are classified as promoters; Immediate downstrea: Peaks that reside within 1000 bases downstream of gene end but not overlap 3 prime UTR are classified as immediate downstream (downstream enhancer)
(D) Heatmap of signal around 1509 TSS regions with H3K4me3 peaks (TSS +- 4000 bp)
(E) The correlation between H3K79Hcy ChIP density in promoter and the gene expression. The ChIP density in promoter were divided to 5 group. The y-axis represents the log2 (RPKM) value. 0-20%: median, 1.99; min, 0; max, 8.35; 25% quantile, 0.60; 75% quantile, 3.11; 20-40%: median, 1.42; min, 0; max, 7.98; 25% quantile, -0.53; 75% quantile, 2.63; 40-60%: median, 1.55; min, 0; max, 7.87; 25% quantile, -0.39; 75% quantile, 2.79; 60-80%: median, 1.54; min, 0; max, 7.86; 25% quantile, -0.66; 75% quantile, 2.83. 80-100%: median, 1.31; min, 0; max, 8.97; 25% quantile, -1.31; 75% quantile, 2.87.
(F) The correlation between H3K4me3 ChIP density in gene body and the gene expression. The ChIP density in gene body were divided to 5 group. The y-axis represents the log2 (RPKM) value. 0-20%: median, 1.31; min, 0; max, 7.63; 25% quantile, -0.52; 75% quantile, 2.48; 20-40%: median, 1.62; min, 0; max, 8.01; 25% quantile, -1.22; 75% quantile, 2.81; 40-60%: median, 1.73; min, 0; max, 7.38; 25% quantile, -0.28; 75% quantile, 2.86; 60-80%: median, 1.80; min, 0; max, 7.98; 25% quantile, -0.33; 75% quantile, 3.04. 80-100%: median, 1.52; min, 0; max, 8.97; 25% quantile, -0.89; 75% quantile, 3.17.

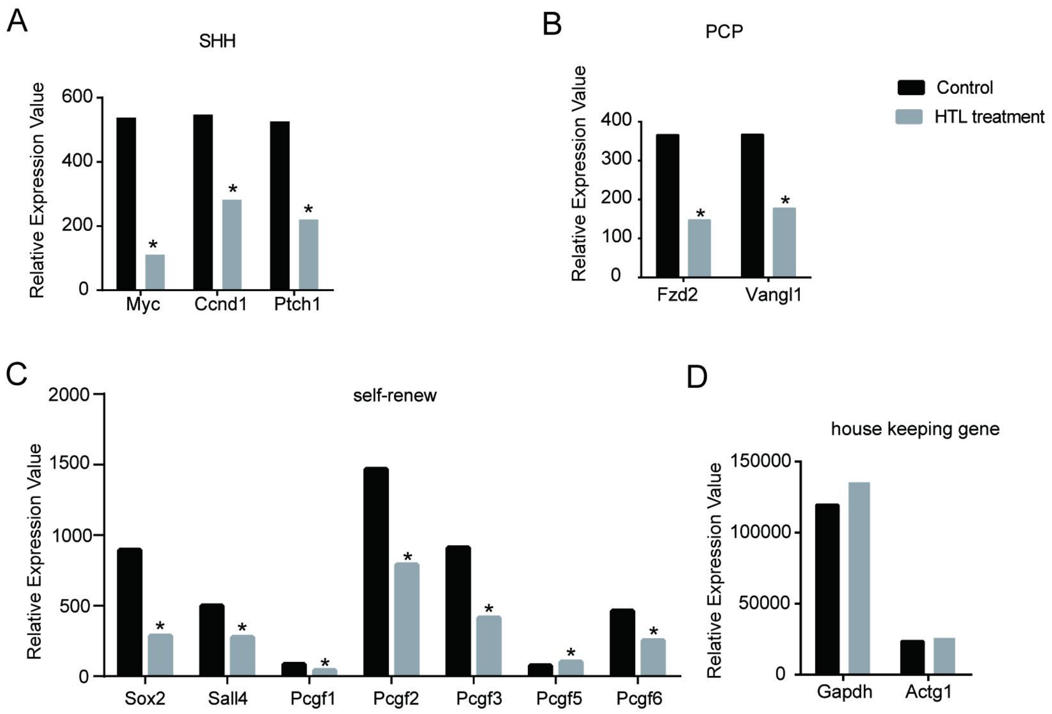

Supplementary Figure 4. Decrease of *Smarca4* gene expression and changes in the expression of other related genes in HTL treated NE4C cells

(A, B, C and D) RT-qPCR analysis of SHH (A) or PCP (B) pathway related genes, self-new related genes (C) and house keeping genes (D)' mRNA expressions levels from NE4C cells treated with or without 0.5mM HTL for 4h. Data represent mean ± SEM (n=3). *$p < 0.05$ versus 0mM HTL treatment; the differentially expressed genes were analysed using Possion Distribution Method.

Supplementary Figure 5. Uncropped blots for Figure 1C-F,

Red boxes indicate the lanes used in the figure.

Fig.1C

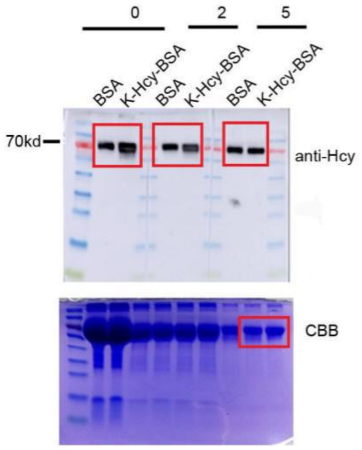

Fig.1D

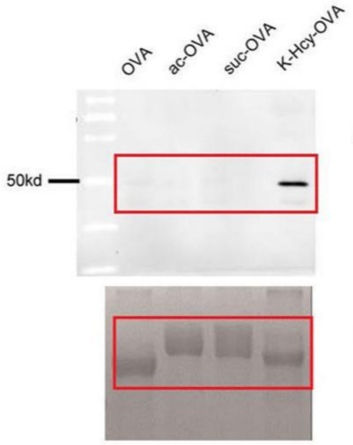

Fig.1E

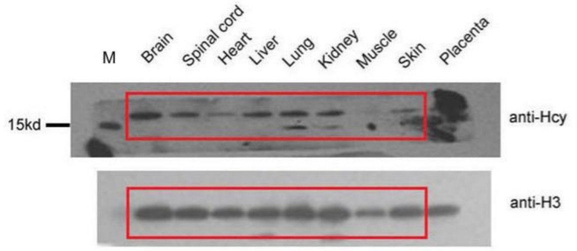

Fig.1F

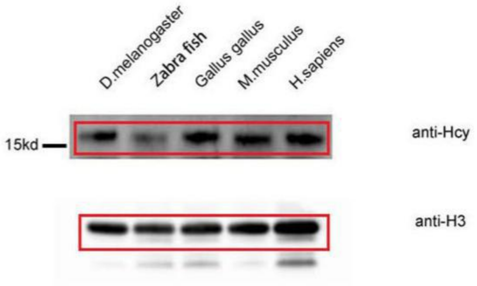

Supplementary Figure 6. Uncropped blots for Figure 2B,

Red boxes indicate the lanes used in the figure.

Fig.2B

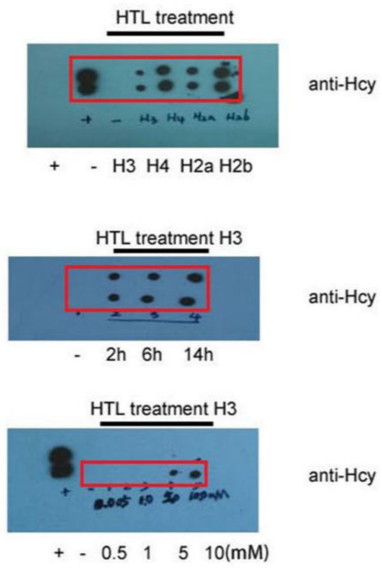

Supplementary Figure 7. Uncropped blots for Figure 3B-C,
Red boxes indicate the lanes used in the figure.

Fig.3B

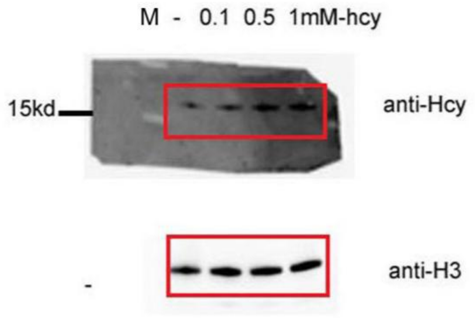

Fig.3C

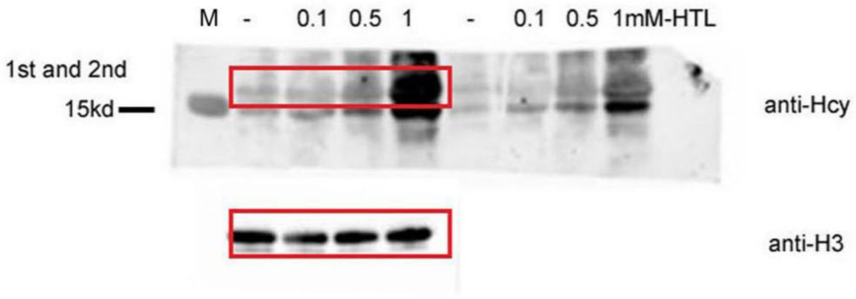

Supplementary Figure 8. Uncropped blots for Figure 4B-D,

Red boxes indicate the lanes used in the figure.

Fig.4B

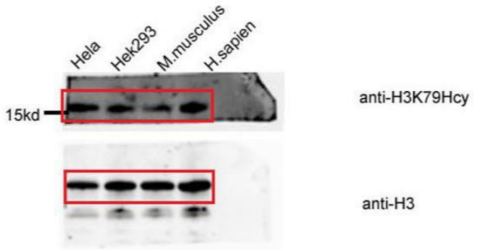

Fig.4C

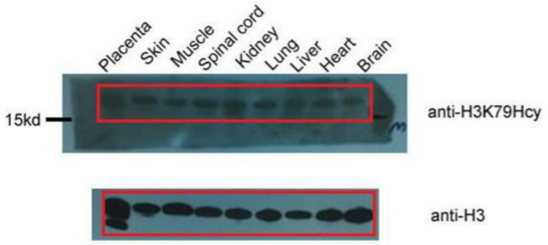

Fig.4D

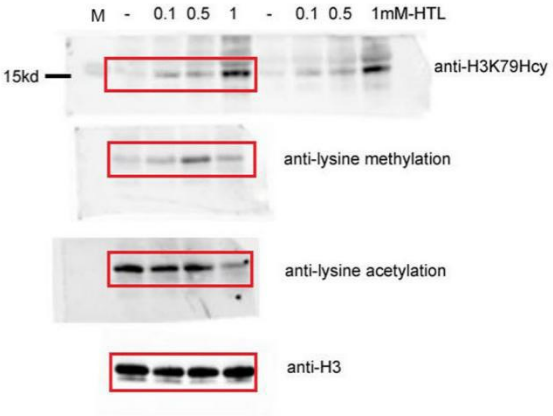

Supplementary Figure 9. Uncropped blots for Figure 5D-E,

Red boxes indicate the lanes used in the figure.

Fig.5D

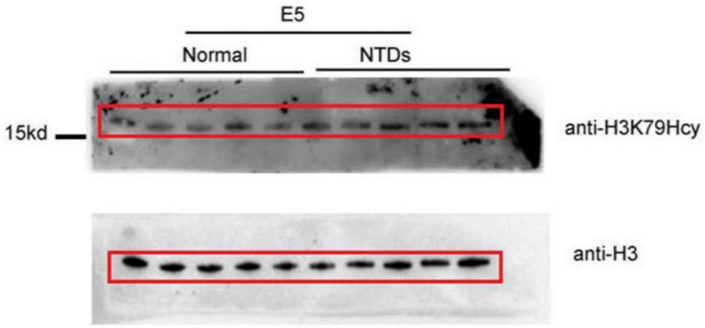

Fig.5E

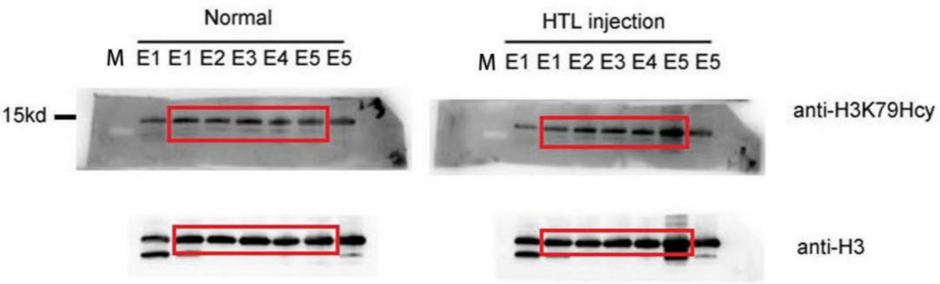

Supplementary Figure 10.Uncropped blots for Figure 7E, Red boxes indicate the lanes used in the figure.

Fig.7E

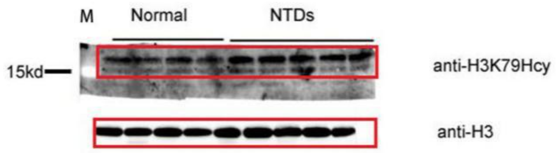

Supplementary Figure 11. Uncropped blots for Supplementary Figure 1A-F,

Red boxes indicate the lanes used in the figure.

Supplementary Figure 1A

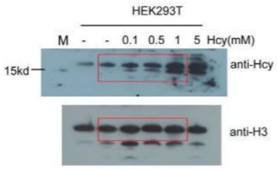

Supplementary Figure 1B

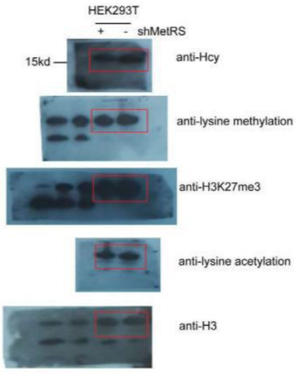

Supplementary Figure 1C

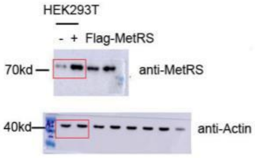

Supplementary Figure 1D

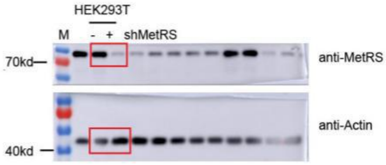

Supplementary Figure 1E

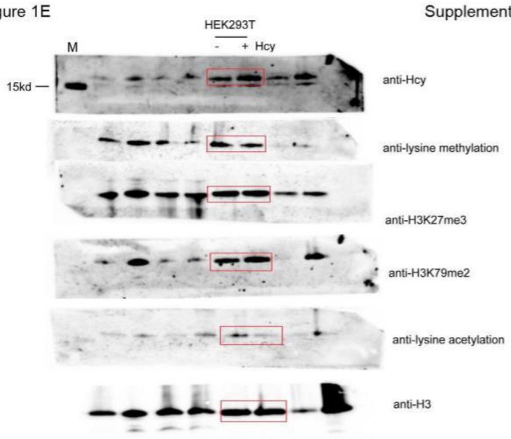

Supplementary Figure 1F

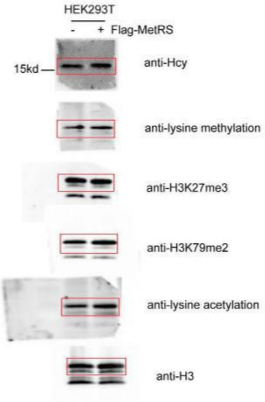

Supplementary Figure 12. Uncropped blots for Supplementary Figure 2A-F,

Red boxes indicate the lanes used in the figure.

Supplementary Figure 2A

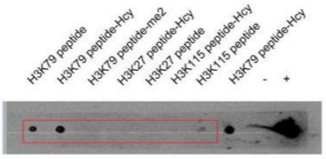

Supplementary Figure 2B

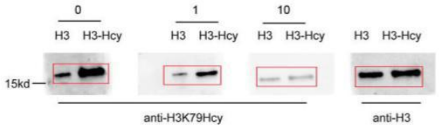

Supplementary Figure 2C

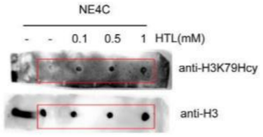

Supplementary Figure 2D

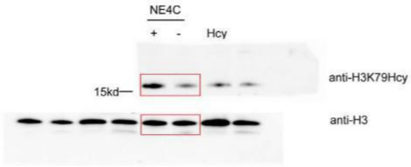

Supplementary Figure 2E-F

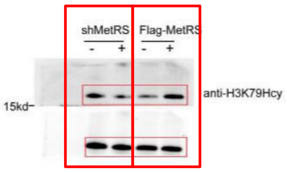

2E 2F

Supplementary Tables

Supplementary Table 1. Clinical manifestations of the human samples.

No.	Source of brain tissue	Hcy (pmol/mg)	FA （ng/mg）	Gender	Gestational age(weeks)
C-1	Normal Control	0.92605	0.0503	Female	39
C-2	Normal Control	3.361722	0.1109	Male	17
C-3	Normal Control	5.542364	0.1812	Female	14
C-4	Normal Control	2.060746	0.0459	Female	20
C-5	Normal Control	4.092798	0.1093	Female	22
C-6	Normal Control	5.501207	0.0578	Female	22
C-7	Normal Control	1.794833	0.0202	Female	25
C-8	Normal Control	2.166592	0.0462	Male	20
C-9	Normal Control	4.375211	0.1885	Male	21
C-10	Normal Control	3.400325	0.0084	Male	22
N-1	Spina bifida	42.48655	0.0698	Female	22
N-2	Closed spinal dysraphism	39.21634	0.1965	Male	22
N-3	Spina bifida	32.76279	0.1185	Female	16
N-4	Spina bifida	31.26128	0.1597	Male	25
N-5	Spina bifida	34.24628	0.1647	Male	24
N-6	Spina bifida	31.63507	0.0851	Female	30
N-7	Spina bifida	45.86487	0.2784	Male	31
N-8	Spina bifida	30.88228	0.1792	Male	20
N-9	Spina bifida	57.5751	0.1436	Female	20
N-10	Spina bifida	64.08609	0.0961	Female	37

Supplementary Table 2. The distribution of histone homocysteinylation in 10 fetal human brain samples

Protein Name	Modification Site	1	2	3	4	5	6	7	8	9	10
H2a	K9	○	●	●	○	●	○	○	○	○	○
	K15	○	●	○	○	○	○	○	○	○	○
	K74	●	●	●	●	●	●	●	●	○	●
	K75	○	●	●	○	●	○	●	●	○	●
	K95	●	●	●	●	●	●	●	●	○	●
	K99	●	●	○	●	●	●	●	●	○	●
	K118	●	●	●	●	●	●	●	●	○	○
	K119	●	●	●	○	●	●	●	●	○	○
H2b	K5	○	●	●	○	○	●	○	○	○	○
	K11	●	●	●	○	●	●	○	○	○	○
	K12	●	●	●	○	●	○	○	○	●	●
	K15	○	○	●	○	●	○	○	●	●	●
	K16	○	○	●	○	●	○	○	○	●	●
	K20	●	○	●	○	●	○	●	○	○	○
	K23	●	○	●	●	●	○	○	○	○	○
	K24	●	○	●	●	●	○	○	○	○	○
	K34	●	○	○	●	●	●	●	●	○	●
	K43	●	●	●	●	●	●	●		○	●
	K46	●	●	●	●	●	●	●	●	○	●
	K57	●	●	●	●	●	●	●	○	○	●
	K108	○	●	●	○	○	○	○	○	○	○
	K116	○	●	○	○	○	○	○	○	○	○
	K120	○	●	●	○	○	○	○	○	○	○
	K125	○	●	●	○	○	○	○	○	○	○
H3	K9	●	●	●	●	●	●	●	○	○	○
	K18	●	●	○	○	●	○	○	○	○	○
	K23	●	●	○	●	●	●	●	○	○	○
	K27	●	●	●	○	●	○	●	●	○	●
	K36	○	●	●	●	●	○	○	○	○	○
	K37	○	●	●	●	●	○	○	○	○	○
	K79	●	●	●	●	●	●	●	●	●	●
	K115	○	●	○	●	○	●	○	○	○	○
H4	K8	○	●	○	○	●	○	○	○	○	○
	K16	●	●	●	○	●	●	○	○	○	●
	K31	○	●	○	○	●	○	○	○	○	○
	K44	○	●	○	○	●	○	○	○	○	○
	K59	●	●	○	●	●	●	○	○	○	○
	K77	○	●	●	●	●	●	○	○	○	●
	K79	○	●	○	○	●	●	○	○	○	●

● the modification in this peptide was detected;

○ the modification in this peptide was not detected.

Supplementary Table 3. Histone peptides including homocysteinylation identified using MS in normal control and HTL treatment NE4C cells

Protein Name	Modification Site	Peptide sequence and modification	
		**HTL treatment**	**Normal control**
H2a	K95	NDEELNK$_{Hcy}$LLGK	QLAIRNDEELNK$_{Hcy}$LLGKVTIAQGGVLPNIQAVLLPK
		DEELNK$_{Hcy}$LLGK	
		HLQLAIRNDEELNK$_{Hcy}$LLGK	
		QLAIRNDEELNK$_{Hcy}$LLGK	
		LNK$_{Hcy}$LLGR	
	K118	PNIQAVLLPK$_{Hcy}$K	
		LPNIQAVLLPK$_{Hcy}$K	
	K119	AQGGVLPNIQAVLLPKK$_{Hcy}$	
		LPNIQAVLLPKK$_{Hcy}$	
		VTIAQGGVLPNIQAVLLPKK$_{Hcy}$	
		GGVLPNIQAVLLPKK$_{Hcy}$	
		PNIQAVLLPKK$_{Hcy}$	
		QGGVLPNIQAVLLPKK$_{Hcy}$	
H2b	K34	SRK$_{Hcy}$ESYSVYVYK	K$_{Hcy}$ESYSIYVYKVLKQVHPDTGISSKAMGIMNSFVNDIFER
	K43	KESYSVYVYK$_{Hcy}$VLK	
		SYSVYVYK$_{Hcy}$VLK	
		SVYVYK$_{Hcy}$VLK	
		YVYK$_{Hcy}$VLK	
		VYVYK$_{Hcy}$VLKQVHPDTGISSKAMGIMNSFVNDIFER	
	K46	K$_{Hcy}$QVHPDTGISSK	KESYSIYVYKVLK$_{Hcy}$QVHPDTGISSK
		VLK$_{Hcy}$QVHPDTGISSK	KESYSIYVYKVLK$_{Hcy}$QVHPDTGISSKAMGIMNSFVNDIFER
		LK$_{Hcy}$QVHPDTGISSK	
	K57	QVHPDTGISSK$_{Hcy}$AMGIMNSFVNDIFER	
	K108	LPGELAK$_{Hcy}$HAVSEGTK	
		LLLPGELAK$_{Hcy}$HAVSEGTK,	
		LLPGELAK$_{Hcy}$HAVSEGTK	
		ELAK$_{Hcy}$HAVSEGTK	
		AK$_{Hcy}$HAVSEGTK	
		K$_{Hcy}$HAVSEGTK,	
	K116	HAVSEGTK$_{Hcy}$AVTK	
		EIQTAVRLLLPGELAKHAVSEGTK$_{Hcy}$AVTKYTS	
	K120	EIQTAVRLLLPGELAKHAVSEGTKAVTK$_{Hcy}$YTS	

H3	K37	TGGVKK$_{Hcy}$PHR	
		ATGGVKK$_{Hcy}$PHR	
		TGGVKK$_{Hcy}$PHR	
	K56	RYQK$_{Hcy}$STELLIR	
		K$_{Hcy}$STELLIR	
		YQK$_{Hcy}$STELLIR	
	K79	AQDFK$_{Hcy}$TDLR	IAQDFK$_{Hcy}$TDLRFQSSAVMA LQEASEAYLVGLFEDTNLCA IHAK
		EIAQDFK$_{Hcy}$TDLR	
	K115	FQSSAVMALQEACEAYLVGLFEDT NLCAIHAK$_{Hcy}$R	
		FEDTNLCAIHAK$_{Hcy}$R	
	K122	PK$_{Hcy}$DIQLAR	
		RVTIMPK$_{Hcy}$DIQLAR	
		MPK$_{Hcy}$DIQLAR	
		TIMPK$_{Hcy}$DIQLAR	
H4	K31	DNIQGITK$_{Hcy}$PAIR	
	K59	LK$_{Hcy}$VFLENVIR	
		GVLK$_{Hcy}$VFLENVIR	
		K$_{Hcy}$VFLENVIR	
	K77	DAVTYTEHAK$_{Hcy}$R	TYTEHAK$_{Hcy}$RK$_{Hcy}$TVTAMDV VYALKR
		VTYTEHAK$_{Hcy}$R	
		YTEHAK$_{Hcy}$R	
		TYTEHAK$_{Hcy}$R	
	K79		TYTEHAK$_{Hcy}$RK$_{Hcy}$TVTAMDV VYALKR
	K91	TAMDVVYALK$_{Hcy}$R	
		TVTAMDVVYALK$_{Hcy}$R	
		AMDVVYALK$_{Hcy}$R	

Supplementary Table 4. Primers used in ChIP assay and RT-PCR

Primer sequence for ChIP assay

Gene		Primers	Primer sequence (5'-3')
Smarca4	Region1	forward primer	TAACACTACACCCAGCTAGTTTCC
		reverse primer	CAGAGCATCTATGTGAGGTCCAT
Smarca4	Region2	forward primer	TAAGGAACAGAAGCCAGGCA
		reverse primer	TGCATTTGTGTGTGAGGGTG
Smarca4	Region3	forward primer	GCCTCTCCGCTCTCTGGAAA
		reverse primer	TTAGCGCTGTGTCATTCTGC
Cecr2		forward primer	CTGTTGGCAGTTTGCTTTCTATT
		reverse primer	CAAGGAACAAGTCAGTAAGGAGC
Dnmt3b		forward primer	GTAGCCTTGAGCTTCCTGTCTGT
		reverse primer	GGCATCTACTTTAGCGAATTGTATT

Primer sequence for RT-PCR

Gene	Primers	Primer sequence (5'-3')
Smarca4	forward primer	CTGCAGCATCACCAGAACAG
	reverse primer	CCACTTCCTTGGGGCTTAGT
Cecr2	forward primer	CCAAGCCGGTTGTGAGTG
	reverse primer	TCTTCCTCACTGCCACTTCC
Dnmt3b	forward primer	CATCAGACAGGGCAAAAACC
	reverse primer	CCGTGTAGTGAGCAGGGAAG
