# Supplementary material for: Elevated H3K79 homocysteinylation causes abnormal gene expression during neural development and subsequent neural tube defects
Source: Nat Commun. 2018 Aug 24;9:3436. doi: 10.1038/s41467-018-05451-7 (PMC6109101; doi:10.1038/s41467-018-05451-7)
Supplement: Supplementary file 3 — Description of Additional Supplementary Files [file 41467_2018_5451_MOESM3_ESM.docx]

**Description of Additional Supplementary Files**

File Name: Supplementary Data 1

Description: Histone peptides including homocysteinylation identified using MS

File Name: Supplementary Data 2

Description: ChIP-seq peak genes identified from H3K79Hcy.

File Name: Supplementary Data 3

Description: ChIP-seq peak genes identified from KHcy.

File Name: Supplementary Data 4

Description: ChIP-seq peak genes identified from H3K4me3.

File Name: Supplementary Data 5

Description: H3K79Hcy DAVID clustering.

File Name: Supplementary Data 6

Description: Gene enrichment on genebody detected by ChIP seq.

File Name: Supplementary Data 7

Description: Gene expression level detected by RNA seq.

File Name: Supplementary Data 8

Description: Histone peptides using MS from in vitro system
